# Reduction of diabetes risk in routine clinical practice: are physical activity and nutrition interventions feasible and are the outcomes from reference trials replicable? A systematic review and meta-analysis

**DOI:** 10.1186/1471-2458-10-653

**Published:** 2010-10-29

**Authors:** Magnolia Cardona-Morrell, Lucie Rychetnik, Stephen L Morrell, Paola T Espinel, Adrian Bauman

**Affiliations:** 1 Sydney School of Public Health, The University of Sydney, Sydney, Australia

## Abstract

**Background:**

The clinical effectiveness of intensive lifestyle interventions in preventing or delaying diabetes in people at high risk has been established from randomised trials of structured, intensive interventions conducted in several countries over the past two decades. The challenge is to translate them into routine clinical settings. The objective of this review is to determine whether lifestyle interventions delivered to high-risk adult patients in routine clinical care settings are feasible and effective in achieving reductions in risk factors for diabetes.

**Methods:**

Data sources: MEDLINE (PubMed), EMBASE, CINAHL, The Cochrane Library, Google Scholar, and grey literature were searched for English-language articles published from January 1990 to August 2009. The reference lists of all articles collected were checked to ensure that no relevant suitable studies were missed. Study selection: We included RCTs, before/after evaluations, cohort studies with or without a control group and interrupted time series analyses of lifestyle interventions with the stated aim of diabetes risk reduction or diabetes prevention, conducted in routine clinical settings and delivered by healthcare providers such as family physicians, practice nurses, allied health personnel, or other healthcare staff associated with a health service. Outcomes of interest were weight loss, reduction in waist circumference, improvement of impaired fasting glucose or oral glucose tolerance test (OGTT) results, improvements in fat and fibre intakes, increased level of engagement in physical activity and reduction in diabetes incidence.

**Results:**

Twelve from 41 potentially relevant studies were included in the review. Four studies were suitable for meta-analysis. A significant positive effect of the interventions on weight was reported by all study types. The meta-analysis showed that lifestyle interventions achieved weight and waist circumference reductions after one year. However, no clear effects on biochemical or clinical parameters were observed, possibly due to short follow-up periods or lack of power of the studies meta-analysed. Changes in dietary parameters or physical activity were generally not reported. Most studies assessing feasibility were supportive of implementation of lifestyle interventions in routine clinical care.

**Conclusion:**

Lifestyle interventions for patients at high risk of diabetes, delivered by a variety of healthcare providers in routine clinical settings, are feasible but appear to be of limited clinical benefit one year after intervention. Despite convincing evidence from structured intensive trials, this systematic review showed that translation into routine practice has less effect on diabetes risk reduction.

## Background

The clinical effectiveness of intensive lifestyle interventions in preventing or delaying development of diabetes in people at high risk has been established from randomised controlled trials of structured, intensive interventions conducted over the past two decades in the USA [1,2], China [3], Finland [4,5], and India[6]. These interventions, promoting healthy eating and moderate physical activity, have shown that sustained weight loss of 3.5 kg or more can be achieved with lifestyle interventions, and that onset rates of diabetes can be reduced by as much as 58% in the first few years. A protective effect of the lifestyle intervention of about 43% has also been shown 20 years following the initial intervention in a Chinese study [7]; and a 34% reduction in diabetes incidence was shown to persist 10 years following an intervention in the USA [8]. There is also evidence, from a large cohort study, that even without a formal intervention, diabetes risk was lowered in people whose lifestyle change was consistent with at least three of the goals of the Finnish Diabetes Prevention program [9]. The study's authors estimated that a further 20% reduction in the incidence of diabetes after 4.6 years of follow-up would occur if a further goal were met.

Calls for broader implementation of lifestyle interventions for diabetes prevention in clinical settings are not uncommon in the literature [10-17], although there has been recognition that translation or replication of randomised controlled trials is not straightforward, and long-term sustainability is uncertain [18,19]. Application of lifestyle recommendations and demonstrated replication of clinical trial approaches in routine clinical practice often are hindered by lack of resources or reimbursement [20], lack of practitioners' time or skill [21,22], practical difficulties with recruitment, measurement error, and poor patient retention due to the complexities of the transition between awareness, motivation and action [18,23,24]. Little systematic information exists on the feasibility or effectiveness of replications of these interventions (or less intensive and more generalisable settings for lifestyle intervention), and on achievement of expected associated benefits as part of routine clinical practice.

To our knowledge, no compilations of trials or reviews of replication studies as part of preventive care in routine clinical practice appear to have been reported. Accordingly, this review presents a summary of outcomes from the routine clinical context and examines the feasibility of transferring the diabetes prevention research to real-world settings. In short, the review assesses the extent that outcomes from clinical trials of lifestyle interventions into physical activity and nutrition to lower diabetes risk have been replicated in routine clinical practice.

## Methods

### Search strategy

**Figure 1:**
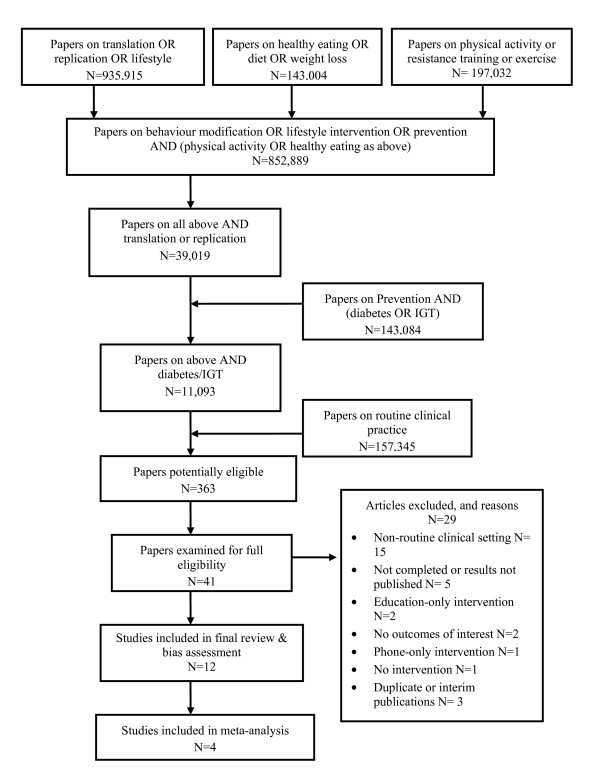
**Summary of search strategy, selection process and outcomes for systematic review, English language papers published 1990-2009**.

The search was confined to English language articles published between January 1990 and August 2009. Three authors (MC-M, LR, AB) separately interrogated different data sources using the same search terms (see appendix). This was supplemented with hand searches of the reference sections of other systematic reviews [2,18,19,25-40]. Only studies which investigated at least one of our research questions above, and which were consistent with our inclusion criteria below, were considered in this review (Figure 1).

### Study selection

The review focused on translational research studies where interventions were based on any of the large reference diabetes prevention RCTs mentioned above. These could be: replication studies in the form of RCTs, before/after evaluations, cohort studies with or without a control group, or interrupted time series analyses, where participants have been exposed to a lifestyle intervention of at least 3 months duration and followed up for at least 3 months. Routine clinical practice was defined as a health service setting providing patient care such as primary health clinics, hospital outpatient clinics or specialist medical centres.

### Intervention types

Interventions were classified as single (either nutrition or physical activity programs with or without medication), or combined nutrition and/or physical activity programs (structured or unstructured) whether or not they included medication. Structured intervention components were defined as those in which participants received a standard set of sessions with instructions on specific dietary and/or physical activity requirements and goals. In unstructured interventions participants were given generic advice on healthy living without specific goals other than improving diet or physical activity in relation to baseline. The comparison group might be 'no intervention group' or an 'alternative intervention' (single or combined). Prevention programs delivering diabetes education materials only were excluded. Likewise, medication-only studies were excluded. Only programs conducted in routine health services, delivered on-site or in associated facilities, with outcomes measured in healthcare settings by general medical practitioners, specialist physicians, practice nurses, dietitians, physiotherapists, allied health professionals, community health staff, or research staff attached to the health service, were included in this review. Interventions either had to be replications or modification of all or some components of the US Diabetes Prevention Program [DPP] [1] or Finnish Diabetes Prevention Study [DPS] [5] or any other reference trial, or had to include the reduction of diabetes risk or diabetes incidence explicitly as a goal or objective.

### Target group

Participants were adult men or women with any degree of impaired glucose regulation (impaired fasting glucose or impaired glucose tolerance) or with normal glycaemia but at risk of diabetes as determined by risk factors such as obesity or family history. They may have been recruited from the primary or other healthcare patient clientele or from the general population but had to receive the intervention through routine healthcare services. Participants' risk of diabetes may have been determined by a diabetes risk score, either measured or from self-report, and may have had accompanying blood glucose tests to either identify impaired glucose regulation or exclude diabetes before receiving the intervention. Studies including patients with diagnosed diabetes were included in this review only if they were a replication of the reference trials and whose outcomes were reported separately from participants without diabetes.

### Outcomes of interest

Studies were included if they reported at least one of the following main outcome measures of interest:

• Improvement in objectively measured risk factors such as weight loss or waist circumference reduction.

• Metabolic outcomes indicative of diabetes risk reduction (improvement of fasting glucose levels, improved 2-hour post-prandial plasma glucose, or reduction of HbA1c)

• Self-reported or objectively measured behavioural outcomes such as increased physical activity (minutes per day or METS per hour), increased fibre consumption (grams per day or gm per KJ), or reduction of fat intake (% of total energy intake).

The secondary outcome examined was:

• Prevention of diabetes (incidence %, or delay in onset or reduction in incidence over a given follow-up time).

### Bias assessment

To assess the potential for bias, and given the heterogeneity of studies included in this review, a generalisability and bias assessment tool covering elements of various checklists and resources from the literature was specifically designed. Items examined included participant recruitment source, selection criteria, treatment allocation, blindness of outcome assessment, simultaneous collection of data for intervention and control groups, measurement error, subgroup analysis and discussion of study limitations (tool available from the authors on request). Reference tools used for this design were STROBE, COCHRANE Collaboration, CLEAR NPT, EQUATOR, PRISMA, TREND and MOOSE [41-46]. One of the authors (MC-M) conducted the bias and quality assessment of all studies and three other authors (LR, SM & PTE) independently conducted the second bias and quality assessment of some of the studies. Two of the authors (MC-M & SM) independently extracted results and assessed the appropriateness of statistical analyses and conclusions.

### Assessment of study quality

Study quality was assessed and graded on the following criteria: (1) evidence of assessment of risk for diabetes at enrolment; (2) explicit eligibility and exclusion criteria; (3) reported participation rate of at least 50% of eligible people; (4) follow-up assessment rates of ≥ 65% of program participants by study conclusion or follow-up; (5) evidence of measurable or explicit outcome assessment; (6) appropriate statistical methods, including adequate control for confounders (in non-RCTs); (7) explicit intervention components; (8) conclusions supported by findings. A numeric score giving equal weight to each of the above criteria was used to determine quality. The maximum possible score was thus 8, indicating highest quality.

### Statistical analysis

The denominator for the effect sizes was the number of subjects in whom the outcome had been assessed. Study results were categorised as positive (statistically significant difference observed), negative (no difference or statistically significant negative effect), or inconclusive (showed no difference but lacked sufficient power to detect a difference). Given the heterogeneity of designs, length of follow-up and outcome measurements of the available studies, pooling of selected results for a meta-analysis was feasible only for four RCTs reporting 12-month follow-up results [47-50]. The remaining eight studies were critically reviewed but not meta-analysed. Changes in means, and tests of heterogeneity between trials were calculated using random effects models. When not reported in individual studies, standard deviations of mean differences in outcome measures were calculated from supplied study participant numbers and standard errors or from 95% confidence limits, either of before-and-after means or from before-and-after differences in mean values. Meta-analysis was conducted using NCSS software version 7.1.1.9 [51] on the four main outcomes of interest: changes in weight, fasting plasma glucose, waist circumference and 2-hour OGTT.

Sensitivity analysis by study quality was not deemed necessary as all four studies finally selected for meta-analysis had a quality score of 7 or 8 out of the possible 8 maximum score. Our search did not identify unpublished replication studies of diabetes prevention in routine clinical practice. Accordingly, we expected findings not to be significantly affected by publication bias.

## Results

Our searches identified 41 potentially eligible diabetes prevention studies of lifestyle interventions in clinical practice that included various combinations of diet and/or exercise for diabetes risk reduction or diabetes prevention. Of these, 18 studies were excluded because: their replications of lifestyle interventions were conducted in non-routine clinical settings (e.g. in community settings such as homes, public centres, churches, or workplaces) [52-59], or in a research setting [47,60-65]; or they did not include at least one of the outcomes of interest [66,67]. A further 5 were excluded because they were trials underway and/or had not published results to date [16,68-70]; or they replicated a reference trial for people who already had diabetes [71]. A further 6 studies were excluded because: the study compared results retrospectively with reference trials without conducting an intervention [9]; the intervention was confined to a diabetes education component only [72,73]; the intervention was telephone-based only and had not replicated components of the reference trials [74]; or they were either duplicates, companion or interim reports, of studies already selected [75-77].

Differences in presentation of results (e.g. monthly weight change without SD [78], or BMI change instead of weight change [79], or FPG ranges instead of group means [79]) precluded inclusion of two studies in the meta-analysis. One study, with the largest sample size [80], could not be meta-analysed to estimate the effects of a lifestyle intervention, as both the medication and placebo arms received the lifestyle intervention, i.e. the study measured the effects of medication as an adjunct to lifestyle intervention.

The final set of 12 studies covered in this review included 7 randomised controlled trials (including one cluster RCT), 3 before-after designs without a control group and two before-after designs with a control group (Table 1). The studies were conducted in 8 OECD countries, and had sample sizes ranging from 58 to 3,304 (median 311), with participant ages ranging from 20 to 79 years; six of the studies targeted middle-aged people only. All interventions combined physical activity and dietary advice, two studies also included medication as part of the intervention [50,80], and all were delivered in routine clinical settings, such as specialist services or hospital outpatient clinics (5), general practitioner consulting rooms (5) or community health services (2). Staff delivering the intervention were usually nurses or allied health staff (8/12). The target groups were people at high risk, defined either by the presence of impaired glucose tolerance, severe obesity, or metabolic syndrome or some of its components. Eight of these studies also included normoglycaemic patients and two replication studies included both subjects with and without diabetes and pre-diabetes.

**Table 1:** Classification of eligible studies by design, target population, outcomes and quality score (1990-2009)

Author, year, reference #	Study Design	Total sample size	Target age group	Country & Setting of recruitment	Inclusion criteria: Normal or abnormal GT	Loss to follow-up rate %	Outcome assessment: Measured OR self-reported	Study Quality score^1^
*Barclay, 2008 [87]	RCT	37	50-85	UK: Single general practice	IGT or IFG	19%	Measured weight, WC, FPG, lipids, self-reported exercise, 4-day food diary	7

Greaves, 2008 [86]	RCT	141	18+	UK: 2 GP surgeries	NGT or IGT	18%	Measured weight, WC and self-reported physical activity	7

*Bo, 2007 [48]	RCT	375	45-64	Italy: Family physician GPs	Metabolic Syndrome	11%	Self-reported FFQ & exercise; Measured FPG, insulin, weight, WC, lipids, CRP	8

*Kosaka, 2005 [49]	RCT	458	Adult males 30+	Japan: Hospital outpatient Clinic	IGT	15.9%	Measured FPG, OGTT, HbA1c, measured weight, lipids	7

Torgerson, 2004 [80]	RCT	3,304	30-60	Sweden: Medical centres	NGT & IGT	57% overall; 48% on medication & 66% on placebo	Measured weight, WC, FPG, lipids, serum insulin, fibrinogen	7

*Dyson, 1997 [50]	RCT	227	40-60	UK, France: 5 Hospitals	IFG	11%	FPG, OGTT, HbA1c, lipids, measured weight, max O2 uptake, self-reported 3-day food record & physical activity log	7

Whittemore, 2009 [78]	CLU	58	21+	USA: Primary care practices	NGT & IGT	12%	Self-reported exercise and nutrition Measured weight, WC, insulin resistance, lipids	7

McTigue, 2009 [84]	BAC	166	20-79	USA: Primary care practices	Obese, NGT or diabetic	7%	Measured weight	5

Eriksson, 1991 [79]	BAC	181	47-49	Sweden: Borderline diabetes clinic	NGT & IGT	22.8%	Self-reported exercise, max O2 uptake, FPG, OGTT, lipids, measured weight, skinfold, mortality	7

Pagoto, 2008 [14]	B-A	118	Middle age	USA: Academic medical center	Metabolic syndrome, NGT or diabetic	17%	Measured weight, BP,	3

Laatikainen, 2007 [83]	B-A	311	40-75	Australia: General practices	NGT & IGT	23.8%	Self-reported FFQ, SF-36, K10; Measured FPG, 2 hr PG, WC, weight, lipids	7

Absetz, 2005 & 2009 [77,82]	B-A	352	50-65	Finland: Primary health care centres	NGT & IGT	9.4%	Self-reported 3-day food diary, physical activity, Measured weight, WC, FPG, lipids	7

BA = before-after; BAC = Before-after with a control group; BP = Blood pressure; CLU = Cluster-randomised trial; FFQ = Food frequency questionnaire; FPG = Fasting plasma glucose; IGT = Impaired glucose tolerance; NGT = Normal glucose tolerance; PA = Physical activity; OGTT = Oral glucose tolerance test; RCT = Randomised controlled trial; WC = Waist circumference.

*Study used for meta-analyses

^1 ^Higher quality score = higher study quality

### Types of lifestyle interventions reported

All studies included a combined lifestyle intervention but two eligible studies included a medication arm in addition to lifestyle. Seven studies attempted replication of the reference trial approaches from either the U.S DPP [1] or the Finnish DPS [81] with adaptation to routine clinical practice, mostly to cater for limitations in practitioner's time and health service budgets [14,48,49,78,82-84]. Modifications included: shorter duration of program (2/7); delivery of group sessions instead of individual face-to-face counselling (4/7); reduced number and frequency of individual or group counselling sessions to which participants were exposed (5/7); and mixed group and individual program approaches (1/7).

Modifications of interventions during the maintenance phase included intermittent support sessions, more economical versions of the resources given to participants, and multidisciplinary teams, either available on site or hired as an additional service. For interventions delivered in a group-based modality, the maximum number of sessions per program was 16, as per the reference trial [1] (median of 6 sessions), but over a shorter period of time. Among the 5 studies reporting delivery of individual counselling sessions, the median number of individual counselling sessions was 13.5.

All dietary interventions were structured and half the physical activity interventions were unstructured (Table 2). While some interventions were delivered with a core intensive phase and an intermittent approach for the maintenance phase, the median duration of intervention was 32 weeks; follow-up periods also varied from 4 to 60 months with a median follow-up duration of 12 months. Delivery of the modified versions of the reference trial interventions was mostly by nurses, psychologists or allied health staff such as health promotion counsellors, dietitians or exercise physiologists alone (8/12) who provided the training, demonstration, counselling or education sessions. Physicians were mainly involved in assessing participant eligibility, referral and outcome measurement (7/12). Two studies did not report the professional background of people delivering the intervention or assessing the participants [49,80].

**Table 2:** Description of studies by lifestyle components and modality of each intervention

	Physical activity program*		Dietary modif-ication*	Face-to-face Counselling		Phone couns-elling	Outcome assessment		Duration (months)		Control Intervention	Program delivered by	Outcomes assessed by
**Author, year [reference]**	**Structured**	**Unstructured**		**No. Individual sessions**	**No. Group sessions**	**No**.	**Objective**	**Self-report**	**Inter-vention**	**Follow-up**			

Barclay, 2008 [87]		Y	Y	-	6	-	Y	Y	1	6	Usual management from GP or nurse	Nutritional scientist, psychologist, aerobics instructor	Nutritionist, research assistant

Greaves, 2008 [86]		Y	Y	11		2	Y	Y	6	6	Usual care + information only	Health promotion counsellor	Researcher

Bo, 2007 [48]		Y	Y	1	4	-	Y	Y	12	12	Usual care + general verbal information	GPs, endocrinolog-ists, nutritionists	Physician

Kosaka, 2005 [49]		Y	Y	1	16	-	Y	Y	12	48	Verbal lifestyle advice every 6 months	NR	NR

Torgerson, 2004 [80]		Y	Y	54	-	-	Y	Y	48	48	Same lifestyle advice minus medication	Dieticians	Doctors & other PHC staff

Dyson, 1997 [50]	Y		Y	5	-	-	Y		12	12	Once only, written basic lifestyle advice	Dietician, fitness instructor, physician	NR

Whittemore, 2009 [78]	Y		Y	6	-	5	Y	Y	6	6	20-30 minutes with nurse & 45 minutes with nutritionist	Nurses	Nurses

McTigue, 2009 [84]		Y	Y		12				12	12	No intervention	Nurses	Physicians

Eriksson, 1991 [79]	Y		Y	7	-	-	Y	-	12	60	No specific diabetes prevention intervention or no intervention	Dietician, Nurse, Physiotherapist	Doctor

Pagoto, 2008 [14]	Y		Y		16		Y		4	4	No control group	Dieticians, psychologists, exercise physiologists	Physicians

Laatikainen, 2007 [83]	Y		Y		6	Y	Y		8	12	No control group	Dieticians, Nurses, Physiotherapist	Other PHC

Absetz, 2005, 2009 [82]	Y		Y		6			Y	8	12	No control group	Nurse, dietician, Physiotherapist	Doctor, Nurse

Y = Yes, reported NR = not reported GPs = general practitioners

* Structured = Participants received standard set of sessions with instructions on specific dietary and/or physical activity requirements and goals;

Unstructured = participants were given generic instructions on improved lifestyle or had flexibility to apply them and no specific goal was set apart from improved diet or physical activity in relation to baseline.

### Type of outcomes reported

Reported measured outcomes of interest were weight (12/12), fasting plasma glucose (9/12) waist circumference (7/12), and 2-hour OGTT (3/12) (Table 3). Six studies had follow-up periods enabling the examination of diabetes incidence or incidence reduction, with the remainder confined to reporting risk improvement via behavioural modification or improvement in metabolic or anthropometric parameters. Self-reported dietary and physical activity outcomes of interest amenable to statistical comparison were not often reported and were confined to mean reduction in fat intake as a percentage of total energy (3/12), and changes in fibre intake (3/12). These are summarised in Table 4. Due to the heterogeneity of units used for measuring and repporting changes in physical activity, it was not possible to meta-analyse these outcomes.

**Table 3:** Results of measured outcomes and direction of effect reported at the end of the study: 1 year follow-up or less, and 3-years or more (1990-2009)

Author, year, reference # follow-up time	Reduction in diabetes incidence (%, OR, RR)	Incidence of diabetes	Improvement of FPG or 2 h PG in mmol/L	% participants achieving ≥ 5% weight loss	Mean weight loss Kg	Mean reduction in WC (cm)
**Results at one year or earlier**						

McTigue, 2009 [84] 1 year	NR	NR	NR	27% of intervention vs. 6% of controls achieved 7% weight loss	-5.2 Kg intervention vs. +0.2 Kg control	NR

Bo, 2007 [48] 1 year	Adjusted OR = 0.23 (0.06-0.85)	1.8% in intervention vs. 7.2% in controls	-0.26 mmol/L FPG intervention vs. +0.07 controls OR for IFG = 0.22 (0.13-0.39)		-0.75 Kg in intervention vs. +1.63 Kg in controls	-2.55 cm in intervention vs. +1.96 cm in controls

Laatikainen, 2007 [83] 1 year	23% based on weight loss; 40% based on WC reduction	2.2% of IGT or IFG participants	-0.14 mmol/L	NR	-2.5 Kg	-4.2 cm

Kosaka, 2005 [49] 1 year	NR	NR	NR	NR	-2.5 Kg in intervention vs.-0.39 Kg in control	NR

Absetz, 2005 [82] 1 year	NR	6% of those meeting 4-5 goals vs. 3% of those meeting 3 or fewer goals	+0.1 mmol/L ± 0.6	12% achieved 5% weight loss	-0.8 Kg ± 4.5 Kg	-1.6 cm ± 4.8 cm

Torgerson, 2004 [80] 1 year	NR	NR	NR	72.8% in medication + lifestyle vs. 45.1% in placebo + lifestyle	-10.6 Kg in medication+lifestyle vs. -6.2 Kg in placebo+ lifestyle	-9.6 cm in medication+lifestyle vs. -7.0 cm in placebo+ lifestyle

Dyson, 1997 [50] 1 year	NR	NR	-0.1 mol/L in intervention vs.-0.2 mmol/L in control	NR	-0.4 Kg in intervention vs. -0.2 Kg in control	NR

Whittemore, 2009 [78] 6 months	NR	N/A	Reported no difference between groups, but no data shown	25% interv vs. 11% control		-0.5 cm intervention vs. -0.1 cm control

Greaves, 2008 [86] 6 months	NR	N/A	NR	23.6% interv vs.7.2% control	Mean difference 1.3 Kg	Mean difference -1.6 cm

Barclay, 2008 [87] 6 months	NR	N/A	-0.02 mmol/L FPG intervention vs. +0.25 mmol/L control at 6 months		-2.73 Kg intervention vs. -0.3 Kg control	-6.01 cm intervention vs. -1.18 cm control

Pagoto, 2008 [14] 4 months	NR	N/A	NR	30% achieved 7% weight loss	-5.5 Kg in whole sample and -6.5 Kg in participants without comorbidities at 4 months	NR

(Laatikainen) Kilkkinen, 2007 [75] 3 months	NR	N/A	No change	NR	-2.4 Kg	-3.2 cm

**Results at 6, 4 or 3 years**						

Eriksson, 1991 [79] 6 years	RR = 0.37 (0.20-0.68)	10% prevalence in intervention vs. 28.6% prevalence in controls	52.2% normalized 2 hr OGTT in intervention vs. 35.7% normalized in IGT non-intervention controls	NR	-3.3 Kg vs. +0.2 Kg	NR

Kosaka, 2005 [49] 4 years	67.4% reduction in intervention group	3% intervention vs. 9.3% in control	53.8% intervention vs. 33.9% in control	NR	-2.2 Kg in intervention vs. -0.39 Kg in control	NR

Torgerson, 2004 [80] 4 years	Total intervention group 37.3%; IGT patients 45%	6.2% in medication + lifestyle vs. 9% in placebo + lifestyle	0.1 mmol/L in medication + lifestyle vs. 0.2 mmol/L in placebo + lifestyle	52.8% vs. 37.3%	-5.8 Kg in medication vs. 3 Kg in placebo	w-6.4 cm in medication + lifestyle vs. -4.4 cm in placebo+ lifestyle

Absetz, 2009 [77] 3 years	NR	12% of those with IGT at baseline vs. 1.2% of those with normal FPG at baseline	0.0 ± 0.8 mmol/L	NR	-1 Kg ± 5.6 Kg	+0.1 cm ± 6.4 cm

NR = not reported N/A = not applicable

**Table 4:** Direction and magnitude of self-reported outcomes at end of program of any duration (1990-2009)

Author, year and reference #	Improvement in frequency of physical activity/week		Reduction of fat intake Mean reduction in energy %		Increased fibre intake in g/day	
	**% achieved goal**	**Mean change**	**% achieved goal**	**Mean reduction**	**% achieved goal**	**Mean increase**

Whittemore, 2009 [78]	+17% in interv vs. +1% in controls at 6 months	NR	NR	NR	NR	NR

Bo, 2007 [48]		+4.73 MET-hr intervention vs. in -0.26 MET-hr in controls at 1 yr		-2.64% in intervention vs. -0.02% in controls at 1 yr		+1.7 g/day intervention vs. +0.17 g/d in controls at 1 yr

Barclay, 2008 [87]	NR	NR	NR	NR	NR	NR

Greaves, 2008 [86]	37.5% interv vs. 27.5% control	NR	NR	Reported successful reduction of total fat and saturated fat but no data shown	NR	NR

Kosaka, 2005 [49]	NR	NR	NR	NR	NR	NR

Torgerson, 2004 [80]	NR	NR	NR	NR	NR	NR

Dyson, 1997 [50]		+0.17 L/min VO2max in interv vs. -0.03 L/min in controls at 1 yr		-3.5% in intervention vs. -1.4% in control at 1 yr		+0.9 g/day intervention vs. -0.7 g/day in control at 1 yr

Eriksson, 1991 [79]	NR	increase of 17% in IGT and 9% Oxigen uptake at 1 yr	NR	NR	NR	NR

McTigue, 2009 [84]	NR	NR	NR	NR	NR	NR

Pagoto, 2008 [14]	NR	NR	NR	NR	NR	NR

Laatikainen 2007 [83]	NR	NR	NR	NR	NR	NR

Absetz, 2005 [82]	66%	NR	48%	NR	52%	

NR = not reported

### Study quality findings

While three of the 12 studies justified their sample sizes on statistical grounds, and not all adjusted for potential confounders, the quality of study design and reporting overall was good in 10 of the 12 studies included, based on quality criteria scores of 7 or 8 out of 8 (Table 1). Two studies were considered suboptimal, with quality scores of 3 or 5 out of 8 respectively [14,84].

Limited generalisability was identified in five studies, where participants recruited were either self-referred healthy volunteers [50] or a convenience sample of males only [49,79], or mostly severely obese middle-age women [14,78]. Two studies reported higher success rates for participants who had already met the goals at baseline [78,82]. In two studies [14,84] the intervention incurred charges and out-of-pocket expenses for each session, which lead to differential exposure to intensity and duration of intervention on the basis of participant's ability to pay. Participation rates for the 8 studies reporting them were satisfactory (median 83.5%). However, in one of the studies, where the participation rate was ostensibly 100%, the control group comprised all those people who did not participate due to financial reasons (on whom outcome measures were collected, but possible exposure to other risk reduction regimes was not recorded) [84].

Loss-to-follow up rates in the 12 studies varied greatly, from 5% to 57% (median 14%). Differential withdrawal rates were reported in a further three studies, where a larger proportion of drop-outs were observed: in participants at highest baseline risk [78]; in those from the intensive arm of the intervention [50]; or in subjects who perceived a poor response to the allocated treatment [80].

### Consistency of findings with reference trials

**Figure 2:**
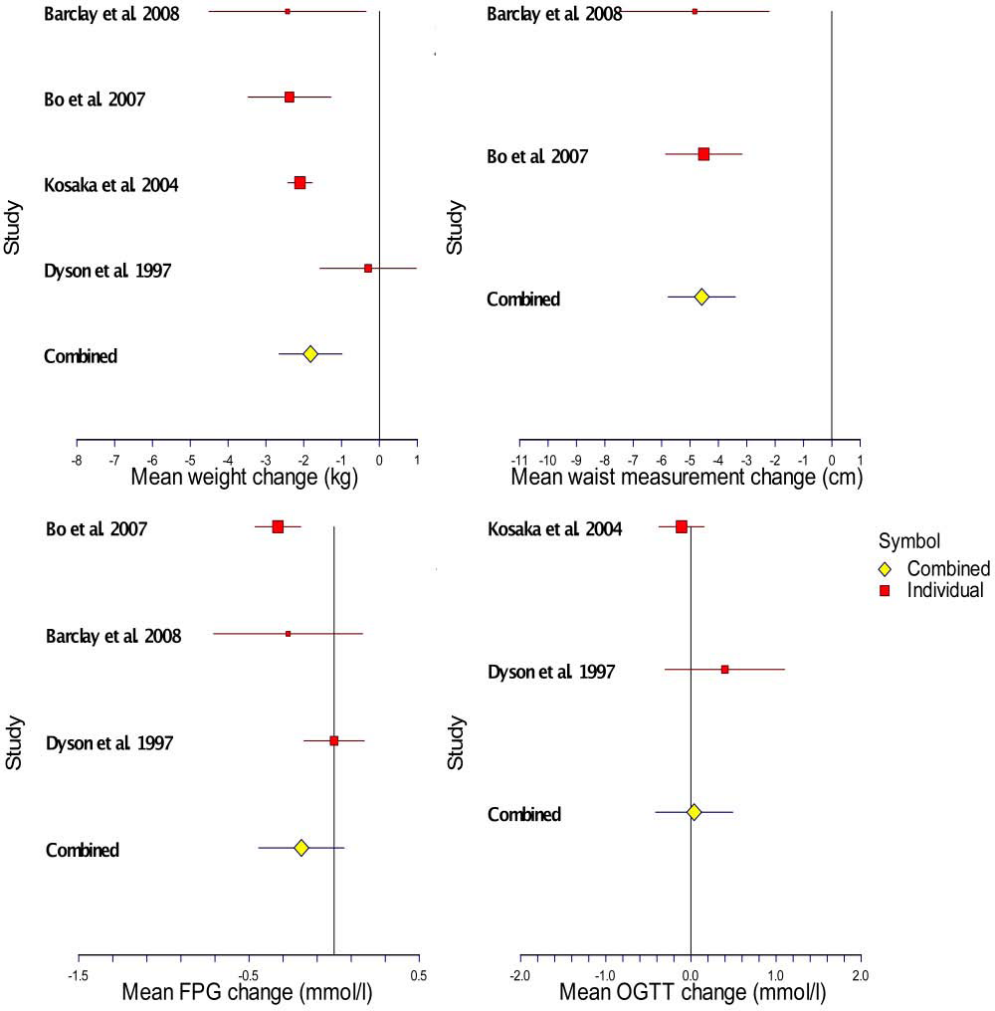
**Meta-analyses and pooled estimates of changes in weight, waist measurement, fasting plasma glucose and 2-hour oral glucose tolerance from selected studies at 1 year follow-up**.

The meta-analysis showed that the pooled weight loss in the intervention group from the four RCTs yielded a weight loss of 1.82 Kg at one year (Figure 2), less than the 5.6 Kg loss observed in the lifestyle-only group of the reference DPP trial or the 4.2 Kg reported in the intervention group of the Finnish DPS. While all studies showed a positive effect on weight loss, only four of the seven studies, 1 RCT and 3 B-A studies [14,79,80,84] reported weight changes at 1 year similar in magnitude to the DPP in the US (around 5 Kg, Table 5). Excepting the XENical in the Prevention of Diabetes in Obese Subjects [XENDOS] trial [80], studies reporting proportions achieving a pre-defined weight loss goal of 5% or 7% were less encouraging. Most studies reported half or less of participants than in the US reference DPP trial, where 50% of participants achieved 7% weight loss at 6 months [1], or in the Finnish DPS where 43% of participants achieved 5% weight loss at 1 year.

**Table 5:** Comparison of selected effect estimates 1 year after the intervention (among studies meta-analysed and not meta-analysed)

Author, year, reference #	Study Type	Effect size for weight*	% achieving a) ≥ 7% or b) ≥ 5% weight loss	% Reduction in diabetes incidence	Effect size for FPG mmol/L	Effect size for 2-hr OGTT mmol/L	Effect for waist circumference (cm)	Effect on fat intake as % of total energy	Effect on fibre intake g/day
**Reference Trials**									

DPP Research Group, 2002 [1]	RCT	-5.6 Kg	a) 49%	NR^§^	-0.3	NR	NR	-6.6%	NR

Finnish DPS [5]	RCT	-4.2 Kg	b) 43%	NR^§^	-0.1	-0.8	-4.4	-21%	-12%

**Meta-Analysed Trials**									

Barclay, 2008 [87]**	RCT	-2.7 Kg		NR	-0.02	NR	-6.01	NR	NR

Bo, 2007 [48]	RCT	-0.75 Kg		NR	-0.26	NR	-2.55	NR	+1.7

Kosaka, 2005 [49]	RCT	-2.5 Kg		0.5%	NR@1 yr	NR	NR	NR	NR

Dyson, 1997 [50]	RCT	-0.5 Kg		NR	-0.1	+0.4	NR	-3.5%	+0.9

**Studies Not Meta-Analysed**									

Greaves, 2008 [86]	RCT	-0.3 Kg	b) 24%	NR	NR	NR	-1.6	NR	NR

Torgerson, 2004 [80]**	RCT	-6.2 Kg		NR	+ 0.2	- 0.4	-7.0	NR	NR

Whittemore, 2009 [78]***	ClustRCT	-1.5 Kg	b) 25%	NR	NR	NR	NR	NR	NR

McTigue, 2009 [84]	BAC	- 5.2 Kg	a) 27%	NR	NR	NR	NR	NR	NR

Eriksson, 1991 [79]	BAC	-5.0 Kg		-37%	NR	-1.5	NR	NR	NR

Pagoto, 2008 [14]	B-A	-5.5 Kg	a) 30%	NR	NR	NR	NR	NR	NR

Laatikainen, 2007 [83]	B-A	-2.5 Kg		-23%	- 0.14	- 0.58	-4.2	NR	NR

Absetz, 2005 & 2009 [77,82]	B-A	-1.0 Kg		NR	+ 0.15	0.0	-1.2 in F +2.3 in M	NR	52% met goal

NR = not reported

References to a) or b) indicate comparison to US DPP or Finnish DPS respectively

* values presented for B-A studies are changes from before to after intervention; values for RCT are differences in change before and after the intervention in the 'lifestyle treatment' group only

§ cumulated diabetes incidence reduction (~58%) reported over 4 and 6 years (US DPP & Finish DPS, respectively)

** values presented as before-after for the lifestyle + placebo group only (excludes effects of medication arm)

*** estimates at 6 months

The one-year improvements in fasting plasma glucose were similar to the DPP across several studies but were too small to be clinically important; and reductions of diabetes incidence in the two studies reporting them at 12 months follow-up [79,83] were somewhat less (37% and 23% respectively) than the reductions apparent from the cumulative risk/incidence plots in the Finnish (~70-80%) and DPP (~70%) trials. For the five studies in this review measuring waist circumference, all concluded that waist circumference reductions were possible with modified lifestyle interventions, but after 1 year only two achieved reductions of sufficient magnitude that cannot be attributed to measurement error (≥ 4 cm) [85]. Decreases in fat consumption and increases in fibre consumption resulting from interventions generally were not reported, and the few studies that did showed no substantial improvements. The exception was the Absetz *et al.* trial which reported half the participants meeting the fibre goal and achieving the total fat intake goal and a third achieving the saturated fat goal [82].

### Feasibility of implementation in routine clinical care

Nine of the 12 studies explored whether translation of the reference trials into clinical care was feasible. Eight concluded that modification of the original trial approaches for adaptation to real life practice made the lifestyle interventions feasible, affordable or replicable in clinical care settings despite barriers to implementation [14,49,78,82-84,86,87]. The remaining study reported that the transferability of the results from original trials to other settings remains questionable, as the positive effect on outcomes diminishes over time [48].

### Meta-analysis results

Seven trials which randomised a total of 4,905 participants to lifestyle intervention or control were identified. The shortest follow-up period was 4 months and the longest follow-up period was six years. Four of these, randomising a total of 1,129 to intervention or control, reported selected outcomes in comparable units at one year [48-50,87]. These were meta-analysed, although not all outcomes of interest were available from all these studies (Figure 2). We chose not to meta-analyse outcomes at four [80] or six years [79], as these relate to the maintenance phase of a program rather than the medium term impact and it would be inappropriate to compare them with one-year results.

The systematic review of RCT results at 12-month follow-up showed: mean weight reduction was 1.82 Kg greater in treatment than control groups which was statistically significant (95% CI:-2.7 to -0.99 Kg); pooled mean waist measurement reductions in treatment exceeded control groups by 4.6 cm, and this was also significant (95% CI:-5.8 to -3.4 cm); fasting plasma glucose reduction was 0.19 mmol/l greater in treatment than controls but non-significant (95% CI: -0.44 to +0.06 mmol/l); and a non-significant greater increase in 2-hour oral glucose tolerance test result of 0.04 mmol/l (95%CI: -0.49 to +0.42 mmol/l). From the above, it is apparent that the interventions can achieve significant weight and waist measurement reductions at one year but do not significantly change the main metabolic indicators of diabetes risk such as FPG or OGTT.

Four of the 12 studies achieved the greatest weight loss, i.e. 5 Kg or more at 12 months. As only two of these successful studies had optimal quality scores [79,80], we further examined the characteristics of these studies to identify common determinants of success in diabetes prevention programs. Common features were RCT design, being based in Sweden, and having long interventions (1 and 4 years) and longer follow-up periods (6 years in Malmo, 4 years in XENDOS). They were not replication studies, and the frequency of participant contact was amongst the highest, with Malmo providing 12 group sessions and XENDOS providing up to 54 individual counselling sessions. Another common feature was that following initial substantial weight loss, the final outcome after several years of follow-up was only an average of 3 Kg weight loss in both studies. We may conclude that the outcomes of these two studies involve social, cultural and health system characteristics unique to that part of Europe that may not be generalisable.

## Discussion

It is apparent that clinical services are making concerted efforts to translate lifestyle intervention trials into routine practice in several countries, whether as pilot studies or as full-scale interventions. All studies included in this review recruited individuals at high-risk of diabetes from IGT, obesity, metabolic syndrome, a combination of these, or based on other standard inclusion criteria. All interventions combined dietary and physical activity and attempted replication of previously published studies. The wide range of intervention intensities, durations of follow-up and outcome assessments reflected the availability of service time, staff skills, levels of reimbursement for prevention services, and limited funding and resources for translation research within the health systems.

Results from the lifestyle intervention studies that relied on weight change show promise in achieving some degree of risk reduction. The weight reduction in intervention subjects exceeded controls by 1.8 kg, which was less than that found in the reference U.S. DPP (5.6 Kg) or the Finnish DPS (4.2 Kg). Results from studies that relied on changes in fasting plasma glucose or 2-hr plasma glucose as a measure of success, were less convincing. However, similarly small changes in FPG after the intervention were also observed in the reference trials (Table 5). Controlled studies meta-analysed here were not successful in showing improved glucose tolerance to a clinically meaningful level that could lead to diabetes prevention.

The independent effects of physical activity and diet and other lifestyle changes in the treatment of pre-diabetes were not examined in many of the studies included in this review. Adjustment for covariates/confounders generally was not conducted or at least not reported in those observational studies we examined. It is possible to combine, 'meta-analytically', outcome measures from observational studies but these must be adjusted for confounding, preferably the same confounding variables measured similarly across studies. We excluded from the meta-analysis all observational studies and some RCT studies due to the heterogeneity of reported outcome measurements [88].

The results from RCTs of routine clinical practice presented here would be expected to occur in a real-world non-experimental setting. However, generalisability from the observational studies examined here is limited given the selection bias of some of the intervention and control groups. The participant population expected through routine care services is 'real-life', self-selected even if programs are offered to all those eligible free of charge. The behaviour of people at risk involves refusals, absenteeism from critical measurement time points and self-selection of healthier and/or more motivated patients. In order to achieve results similar to the RCT evidence, these practical issues of non-compliance would need particular attention in a real world setting. The Diabetes in Europe - Prevention using Lifestyle, Physical Activity and Nutritional intervention (DE-PLAN) [89] is developing the structures for a prevention management model which can be implemented in routine clinical practice settings. Results from this project should shed further light on specific success factors for research translation.

This review also examined the feasibility of implementation of interventions as an integral part of routine clinical care, as this can inform policy on dissemination of diabetes prevention programs or associated subsidies within healthcare systems. To this end, we examined authors' conclusions on whether the given intervention could sustainably be incorporated into usual care provided, for example, without the need for excessive time beyond usual consultation, additional funding or contracting of external staff.

Finally, while the outcomes of the two US studies, where participation incurred a fee, probably are the most representative of real life in USA, such market-based rationing of diabetes prevention might not be acceptable in other health systems, and certainly would not reach those most in need of such interventions, including low socio-economic groups and people with higher prevalence of risk factors for diabetes.

### Strengths of this review

To our knowledge, this is the first attempt to comprehensively compile feasibility and effectiveness of translation of diabetes prevention trials specifically for routine clinical settings.

We used a purpose-built comprehensive quality scoring system based on individual components of relevance from checklists widely used by others in quality assessment of the literature. Our quality criteria allowed for the inclusion of several study types to maximise the chances of identifying and assessing relevant diabetes prevention programs. The search was extensive and individual study authors were contacted to either confirm that their study was conducted under routine clinical care or to exclude any translation study conducted in research settings or under simulated clinical care. Meta-analytic techniques were used when feasible.

### Limitations

Despite the good quality of papers covered in this review, the total number of studies finally included was small; some were exploratory (3 pilots) and many of them had short follow-ups and only modest sample sizes which essentially reflect the financial and time restrictions of real-life interventions in routine clinical practice. We included studies with intervention and follow-up durations of at least 3 months. These are not unusual in routine practice, as modifications to duration and intensity of the strict approaches in the reference trials are common in the replication literature. While longer interventions and follow-up times are ideal, in real-world situations longer studies inevitably are affected by sample attrition and attendant generalisability issues. We wanted to include some measurement of short-term impact and avoid attrition bias and selection bias in our assessment of what is being evaluated in routine practice and therefore we allowed for feasibility and pilot studies to be incorporated.

Analyses from before-and-after studies often did not report on adjustment for confounders. More importantly, the reporting of outcomes of interest was often incomplete or in disparate units of measurement precluding inclusion in the meta-analysis. However, the overall good quality of these studies enabled their inclusion in the broader systematic review.

Many weight-loss-only programs and other lifestyle interventions for reduction of cardiovascular disease risk were excluded as they did not specifically mention replication of the diabetes prevention trials. However, we acknowledge that results from these may also be applicable to diabetes risk reduction, and while examples of reviews of these are available in the literature, their focus is beyond the scope of our review.

## Conclusions

Despite convincing evidence from structured intensive randomised controlled trials in research settings, this systematic review shows that translation into routine practice has somewhat less of an impact on diabetes risk reduction. Given the heterogeneity and limitations of the studies included in this review, it is also not possible to determine conclusively whether the type of clinical setting, the frequency or intensity of interventions, or the modality of the intervention (face-to-face, telephone, written materials, etc) are critical success factors for translation of diabetes prevention programs in routine clinical care. Nor was it possible to assess the separate contributions of individual lifestyle change components to diabetes risk reduction. Accordingly, we cannot yet make specific recommendations on the most effective features of these targeted lifestyle interventions.

However, based on our findings, the direction of the effects on the four most commonly reported outcomes (weight, FPG, waist circumference and 2-hour OGTT) are encouraging; and the consensus on feasibility of their modification as part of routine care without excessive cost suggest that it is still worth promoting the translation of modified, group-based lifestyle interventions, and conducting more rigorous evaluations in these settings. The establishment of a register of translation projects using consistent, measurable outcomes would add more certainty to the effectiveness of routine practice interventions, and when more studies with larger sample sizes and data on intermediate end-points become available they could be included in a more comprehensive meta-analysis.

## Appendix - Description of the search strategy

### Electronic sources searched

• Articles were identified through searches in MEDLINE, PubMED, The Cochrane Library, Google Scholar, CINAHL and EMBASE.

• Internet searches and searches of the grey literature were conducted to identify non peer-reviewed internal reports from government and health services websites and non-government sources.

### Supplementary sources

• Hand searches of reference lists from related articles found whether or not they were eligible for inclusion in this review

• Hard copy Australian government publications and unpublished internal reports from key informants for non-indexed publications.

• Authors of reviewed articles were contacted by MC-M if it was unclear from their papers whether the intervention was conducted in a research or community-based or a routine clinical setting. However, due to resource constraints, no attempt was made to contact the investigators whose papers did not report all measured outcomes.

### Search terms

Diabetes, Pre-diabetes, Type 2 diabetes, Impaired glucose tolerance OR glucose intolerance, Lifestyle intervention OR lifestyle program OR strategy, Physical activity OR Exercise OR Resistance Training, Healthy eating OR diet OR dietary modification OR weight loss, Behavioural modification, AND (Primary health care, General practi$., clinical practice, routine clinical care), AND (Prevent$. Ti, ab, Translating OR Translation OR Translat$. Ti, ab., Translation research OR translational study OR Replication study).

## Competing interests

The authors declare that they have no competing interests.

## Authors' contributions

MC-M, conceived the study, designed the quality and bias assessment tools, conducted database searches and quality assessments, wrote the first draft of the manuscript and incorporated changes suggested by others. MC-M coordinated database searches by LR and AB; LR, SM and PTE conducted bias assessments; AB and LR assisted in the design of the study; MC-M and SM performed the statistical analyses. SM, AB and LR helped to comment on and refined the manuscript. All authors have read and approved the final manuscript.

## Pre-publication history

The pre-publication history for this paper can be accessed here:

http://www.biomedcentral.com/1471-2458/10/653/prepub
